# Influence of iron uptake systems on cefiderocol activity in *Escherichia coli*: At the crossroads of antibiotic resistance and virulence

**DOI:** 10.1128/aac.01679-25

**Published:** 2026-07-10

**Authors:** Emilie Mallecot, Valentine Berti, Marie Petitjean, Julie Meyer, Signara Gueye, Thibaut Morel-Journel, Sarah Timsit, Olivier Clermont, Laurent Poirel, Erick Denamur, Guilhem Royer, Laurence Armand-Lefevre

**Affiliations:** 1 Assistance Publique-Hôpitaux de Paris (AP-HP), Hôpital Bichat-Claude Bernard, Laboratoire de Bactériologie 55076 , Paris, France; 2 Université Sorbonne Paris Nord et Université Paris Cité, INSERM, IAME 27102, Paris, France; 3 Faculty of Science and Medicine, Medical and Molecular Microbiology, University of Fribourg 27211, Fribourg, Switzerland; 4 Swiss National Reference Center for Emerging Antibiotic Resistance (NARA), University of Fribourg, Fribourg, Switzerland; 5 Assistance Publique-Hôpitaux de Paris (AP-HP), Hôpital Bichat-Claude Bernard, Laboratoire de Génétique Moléculaire 55076 , Paris, France; 6 Département de Prévention, Diagnostic et Traitement des Infections, Assistance Publique-Hôpitaux de Paris (AP-HP), Hôpital Henri Mondor 378967 , Créteil, France; 7 Université Paris-Est Créteil, IMRB, INSERM U955, Équipe "Virus, Hépatologie, Cancer", Créteil, France; Universita degli Studi di Roma La Sapienza, Roma, Italy

**Keywords:** *Escherichia coli*, NDM, Enterobacterales, cefiderocol, iron uptake system

## Abstract

Cefiderocol (FDC) is a new siderophore-conjugated cephalosporin that enters the periplasm *via* iron transport systems. However, the specific contribution of individual iron uptake pathways to FDC activity remains unclear. We investigated the role of 13 iron acquisition systems using several *Escherichia coli* strain collections. FDC MICs were determined in iron-depleted and iron-supplemented media for *E. coli* mutants (Keio and pathogenic island [PAI]-deleted collections) and for no-acquired β-lactamase or TEM (NoBL/TEM-*Ec*) and NDM-producing *E. coli* (NDM-*Ec*) clinical isolates. Prevalence of iron uptake genes was assessed in these isolates, and *fec* operon prevalence and genomic location were investigated in *E. coli* genomes from EnteroBase and RefSeq databases. Compared to K-12 and 536 reference *E. coli*, only Δ*cirA* and Δ*fiu* (enterobactin system) showed increased FDC MICs (8- and 3-fold, respectively), while Δ*fecA* and Δ*fecB* had lower MICs (3-fold decrease). The *fec* operon, a known extraintestinal virulence factor, was significantly more prevalent in isolates with FDC MICs above median than below (96% vs. 33% in NoBL/TEM-*Ec*; 100% vs. 0% in NDM-*Ec*). According to EUCAST breakpoints, 63.6% of *fec*-positive NDM-*Ec* were resistant to FDC, whereas none of the *fec*-negative were. The *fec* operon was found in 46.5% of *E. coli* genomes, including virulent clones (ST131, 77%) and was mostly chromosome-borne (99%). Plasmid-borne *fec* closely resembled that of *Enterobacter hormaechei or Klebsiella pneumoniae*, suggesting interspecies transfer. Our findings highlight the role of Fec in reducing FDC susceptibility and promoting resistance in NDM-producing *E. coli*. They challenge the virulence-resistance trade-off demonstrating the “liaisons dangereuses” between iron and antibiotics.

## INTRODUCTION

Since the 2000s, the emergence of carbapenemase-producing Enterobacterales (CPE) has become a global public health threat (1). CPE are resistant not only to last-line β-lactams but also to almost all other classes of antibiotics, leaving few therapeutic alternatives in case of infection. Among these, metallo-β-lactamase (MBL)-producing Enterobacterales, that include NDM and VIM enzymes, are of particular concern as they represent a major therapeutic challenge due to the limited treatment options.

Cefiderocol (FDC) is a novel class of β-lactam, corresponding to a siderophore-conjugated cephalosporin molecule active on difficult-to-treat Gram negative bacilli including CPEs (2). The chemical structure of FDC is close to cefepime and ceftazidime and contains a chlorocatechol moiety resembling catecholate siderophore. Its activity is based on a “Trojan horse” strategy that uses active catechol uptake systems by mimicking siderophore molecules to facilitate the entry of FDC into the periplasmic space. Once inside, FDC binds to penicillin-binding proteins (PBP) and then inhibits peptidoglycan synthesis. Therefore, FDC has become a valuable option in the treatment of infections caused by CPE including those that produce MBL.

Under physiological conditions, free iron concentrations in human fluids are extremely low (~10^−24^ M as ferric ion Fe^3+^) (3). Iron is mainly present as an insoluble form and is strongly bound to proteins such as transferrin to limit its toxicity and bacterial proliferation. It is, therefore, essential for bacteria to synthesize siderophores and iron uptake systems to obtain the iron needed to survive and multiply in iron-deficient conditions (4). Iron uptake systems are, indeed, considered to be among the most important virulence factors in bacteria, as they are essential for bacterial multiplication *in vivo. Escherichia coli* has numerous iron uptake systems enabling transport of Fe^3+^, Fe^2+^, and heme. Fe^3+^ transport systems include siderophores such as enterobactin (Ent), salmochelin (Iro), yersiniabactin (Ybt), and aerobactin (Iuc) associated with receptors FepA, Fiu, CirA, IutA, IroN, and FyuA, high affinity iron transporters such as the ferric di-citrate transporter FecA and the ferrichrome transporter FhuA/FhuE. Fe^2+^ is incorporated through non-specific porins (OmpC/OmpF) and transported by the Feo, Efe, and Sit systems. Finally, heme uptake systems involve ChuA and Hma (5, 6).

Since its introduction, several mechanisms associated with decreased susceptibility or resistance to FDC have been reported. Certain β-lactamases, particularly MBLs such as NDM, have been linked to reduced FDC activity, with increased MICs reaching up to 256 mg/L (7, 8). Mutations in genes encoding iron acquisition systems, notably *cirA* and *fiu,* have also been described and are associated with increased FDC MICs (9, 10). However, the influence of siderophores and other iron capture systems in *E. coli* has not been thoroughly investigated. In this study, we aimed to elucidate the role of various iron uptake systems in FDC activity using (i) well-characterized collections of *E. coli* mutant strains lacking β-lactamase production and (ii) a collection of clinical isolates producing multiple acquired antibiotic resistance mechanisms, including MBL-producers.

## MATERIALS AND METHODS

Several collections of strains were used in this study

### Keio knockout collection

The Keio collection is a library of single-gene deletion mutants derived from *E. coli* K-12 strain BW25113, a commensal strain from phylogroup A, exhibiting a wild-type antibiotic susceptibility pattern (11). For this study, 18 mutants with deletions in genes involved in iron uptake (Δ*cirA*, Δ*fiu*, Δf*epA*, Δ*fepB*, Δ*fepC*, Δ*entB*, Δ*fhuE*, Δ*fhuB*, Δ*fhuD*, Δ*fecA*, Δ*fecB*, Δ*fecC*, Δ*ompF*,Δ*ompC,* Δ*efeU*, Δ*efeO*, Δ*feoA*, Δ*feoB*) were selected. The wild-type K-12 BW25113 strain (K-12-WT) was used as control. The presence of the kanamycin resistance cassette in Keio mutants was confirmed by PCR using specific primers (Thermo Fisher Scientific, Waltham, MA, USA), followed by gene-specific PCRs to verify each deletion; control PCRs were also performed on the K-12-WT strain.

### 536-PAI-deleted mutants

A collection of mutants derived from the uropathogenic *E. coli* 536 strain from the phylogroup B2 (12) was previously constructed by single or multiple deletions of pathogenicity-associated islands (PAIs) (13). For this study, three mutants with the deletion of PAI that include genes involved in iron uptake systems were selected: ΔPAI-III (salmochelin and heme receptor HmuR), ΔPAI-IV (yersiniabactin), and PAI-dTTP (all PAIs deleted). The wild-type *E. coli* 536 strain (536-WT) was used as the control.

### *E. coli* clinical isolate collections

The clinical isolates included 65 non-duplicate *E. coli* isolates either lacking acquired β-lactamase or carrying the narrow-spectrum penicillinase TEM-1 (NoBL/TEM-*Ec*), previously characterized by Royer et al. (14) and 24 non-duplicate and non-clonal NDM-1-producing *E. coli* (NDM-*Ec*) isolated from patients hospitalized at the Bichat-Claude Bernard University Hospital. NDM-*Ec* were mainly from rectal or throat screening samples (*n* = 20), the remaining were from urinary (*n* = 2), respiratory (*n* = 1), and blood (*n* = 1) samples. These NDM-*E*c isolates were selected according to their plasmid harboring the *bla*_NDM-1_, which were either IncFK-pNDM_Epi1 (*n* = 15) or IncC-pCMY-4 (*n* = 9), already described (15) or available in Supplementary Materials. Short- and long-read whole-genome sequences of all new clinical isolates are available at Bioproject PRJNA1432676.

### Cefiderocol susceptibility testing

Minimum inhibitory concentrations (MICs) of FDC were determined using the UMIC Cefiderocol kit (Bruker, Brandol, France) in iron-depleted cation-adjusted Mueller Hinton Broth (ID-CAMHB, Bruker) according to the European Committee on Antimicrobial Susceptibility Testing guideline (EUCAST https://www.eucast.org/). Each MIC was determined at least twice. Additional measurements were determined if the results differed by more than one dilution. Except for the *ΔompC* mutant, all MICs performed in iron-depleted media were determined simultaneously and using the same reagent batch number.

### Cefiderocol susceptibility testing in iron-supplemented MH broth

To assess the impact of iron concentration on FDC MIC, 10 strains from the Keio (Δ*cirA*, Δ*fiu*, Δ*fepA*, *ΔentB*, Δ*fecA*, Δ*ompF*, Δ*ompC*) and PAI (ΔPAI-III, ΔPAI-IV, and PAI-dTTP) collections were selected as well as both wild-type strains (K-12-WT and 536-WT). ID-CAMHB was enriched with ammonium iron (III) citrate (F-5879-100G, Sigma-Aldrich, France) to reach 9 iron citrate concentrations: 0.01, 0.05, 0.1, 0.5, 1, 5, 10, 50, and 100 mg/L corresponding to 0.03, 0.16, 0.31, 1.57, 3.13, 15.67, 31.34, 156.68, and 313.37 µM of iron, respectively. FDC MIC of each strain was determined in duplicate at each of the 9 iron concentrations. MICs in iron-supplemented media were determined for each mutant using the same reagent batch number.

### Search for iron uptake systems on genomes

When analyzing genomes of clinical isolates, resistance and virulence genes were searched using Abricate v1.0.0 using ResFinder4 and a custom database, respectively. Identity and coverage thresholds were set at 70% and 90%, respectively, to avoid missing detection of divergent alleles. The percentage of coverage and identity as well as the start and end positions of the alignment relative to the gene length are provided in Table S2. The custom database included genes or operons related to iron uptake system, namely, *chu*, *hmA*, *ent*, *cirA*, *fiu*, *fep*, *fes*, *ire*, *iro*, *irp*, *ybt*, *fyuA*, *iuc*, *iut*, *fhu*, *fec*, *ompC*, *ompF*, *efe*, *feo*, *sit*, *tonB*, *exb*, *baeS*, *envZ*. Sequences were found in the NCBI (https://www.ncbi.nlm.nih.gov) and UniProt (https://www.uniprot.org) database (Accession numbers available in Table S1). Disruption or mutation in *cirA* and *fiu* genes leading to truncated protein were searched by alignment of each protein sequences with its wild-type.

Additionally, the presence of the *fec* operon was screened in the genomes of the 70,301 *E. coli* strains indexed in Enterobase (https://enterobase.warwick.ac.uk/) (16, 17) and the complete genomes of 2,302 *E. coli* strains available in RefSeq (https://www.ncbi.nlm.nih.gov/refseq) (14). The location of the *fec* operon (plasmidic or chromosomal) was determined for the genomes of *E. coli* from RefSeq and of three clinical NDM-*Ec* isolates, all generated using long-read approach. Plasmid typing was performed using Abricate with the Plasmidfinder database (18) applying identity and coverage thresholds of 80%. Finally, the genetic environment of the *fec* operon in all plasmid sequences carrying a complete operon (*fecIRABCDE*) was analyzed and compared with Clinker (19) using a 20-kbp region downstream and upstream of *fecA,* including the plasmid previously described by Polani et al. (20) (accession number JN233704.1).

### Statistical analysis

R (https://www.R-project.org/) and RStudio software (Posit Software, PBC) were used to perform biostatistics analysis and to create graphic representations. FDC MICs of each Keio and 536-PAI-deleted mutant were compared to corresponding wild-type strains, K-12-WT and 536-WT, respectively, using Wilcoxon test. The effect of iron on the FDC MICs of the selected mutants was analyzed and represented by the LOESS (Locally Estimated Scatterplot Smoothing) regression method. Fisher tests were carried out to determine the significant associations between the iron transport genes and the MICs of the clinical strains. *P*-values < 0.05 were considered statistically significant.

For each of the NoBL/TEM-*Ec* and NDM-*Ec* collections, a principal component analysis (PCA) was performed on a matrix of presence in each isolate of operons related to the iron uptake systems listed above. An operon was considered present (noted 1 in the matrix) if all its genes were detected, and absent (noted 0) otherwise. For each analysis, the set of principal components explaining more than 90% of the observed variance were used as the explanatory factors of a linear regression on the Log_2_ of the observed MICs. These two regressions provided estimates of the change in MIC along the principal components for the isolates of the two collections.

## RESULTS

### Cefiderocol activity on Keio and 536-PAI-deleted mutants

**Fig 1:**
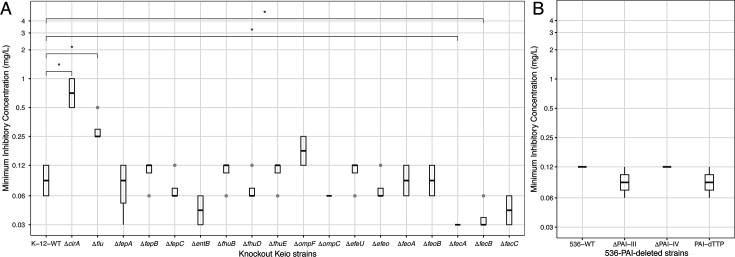
Cefiderocol (FDC) minimum inhibitory concentrations (MIC) of Keio and 536-PAI-deleted mutants in iron-depleted media (ID-CAMHB) for (**A**) 18 Keio mutants (Δ*cirA*, Δ*fiu*, Δ*fepA*, Δ*fepB*, Δ*fepC*, Δ*entB*, Δ*fhuB*, Δ*fhuD*, Δ*fhuE*, Δ*ompF*,Δ*ompC*, Δ*efeU*, Δ*efeO*, Δ*feoA*, Δ*feoB,* Δ*fecA*, Δ*fecB*, Δ*fecC*) and (**B**) 3 PAI-deleted mutants (ΔPAI-III, ΔPAI-IV, PAI-dTTP). MICs median for each mutant was compared to those of the K-12-WT (**A**) or 536-WT (**B**) strains. * indicates *P* < 0.05 according to a Wilcoxon test.

The median FDC MIC of the K-12-WT was 0.09 ± 0.04 mg/L. Among the 18 single-deleted mutants from the Keio collection, only four showed an MIC to FDC that was significantly different from that of the K-12-WT strain. The MIC of Δ*cirA and*Δ*fiu* mutants were 0.75 mg/L and 0.25 mg/L, respectively, representing eight- and threefold higher values compared to the K-12-WT (*P* = 0.025 and 0.027). Conversely, the MICs of the Δ*fecA* and Δ*fecB* mutants were both 0.03 mg/L, corresponding to a 3-fold lower value relative to K-12-WT (*P* = 0.019 and 0.047). Although not statistically significant, the MIC of the Δ*ompF* (0.19 mg/L) tended to be higher than that of the K-12-WT strain (*P* = 0.086) (Fig. 1A).

Median FDC MICs of the ΔPAI-III (salmochelin and heme receptor HmuR), ΔPAI-IV (yersiniabactin), and PAI-dTTP (all 536-PAI-deleted) mutants ranged from 0.06 to 0.125 mg/L with no difference compared to the 536-WT strain (Fig. 1B).

### Cefiderocol activity on Keio and 536-PAI-deleted mutants with increasing iron concentration

**Fig 2:**
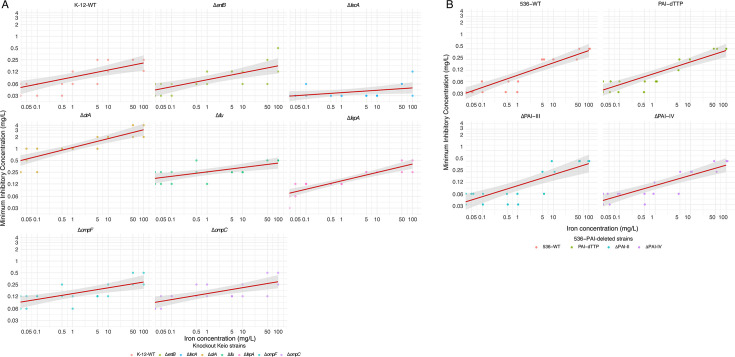
Locally Estimated Scatterplot Smoothing (LOESS) representation of Cefiderocol (FDC) minimum inhibitory concentrations (MIC) of Keio (**A**) and 536-PAI-deleted (**B**) mutants with increasing iron concentration. FDC MICs were measured in ID-CAMHB media supplemented with nine iron concentrations: 0.01, 0.05, 0.1, 0.5, 1, 5, 10, 50, and 100 mg/L (corresponding to 0.03, 0.16, 0.31, 1.57, 3.13, 15.67, 31.34, 156.68, and 313.37 µM of iron, respectively). MICs were determined for (**A**) seven Keio mutants (Δ*entB*, Δ*fecA,*Δ*cirA*, Δ*fiu*, Δ*fepA*, Δ*ompF*, Δ*ompC*) and (**B**) three 536-PAI-deleted mutants (ΔPAI-III, ΔPAI-IV, PAI-dTTP) at each iron concentration. K-12-WT (**A**) or 536-WT (**B**) strains were tested as control.

The influence of the different iron uptake systems under increasing iron concentrations was measured for selected mutant strains using ID-CAMHB supplemented with iron (0–100 mg/L). The Δ*cirA*, Δ*fiu,* and Δ*fecA* mutants were included on the basis of significant MIC changes that were observed in iron-depleted media. The Δ*fepA* mutant was included to assess all receptors of the enterobactin system. The Δ*entB* mutant, deficient in a catecholate-type siderophore naturally produced by *E. coli*, was also included to assess a potential competition between enterobactin and FDC. All 536-PAI-deleted mutants were tested to evaluate the contribution of the salmochelin and yersiniabactin systems. Finally, the Δ*ompF* and Δ*ompC* mutants were tested due to the slightly decreased FDC susceptibility of Δ*ompF* in iron-depleted conditions despite not being directly involved in siderophore-mediated iron uptake but rather enabling the diffusion of FDC. The FDC MICs of all tested strains significantly increased with rising iron concentrations (*P* < 0.01) except for ∆*fecA* (*P* = 0.056) (Fig. 2A and B and Table S3). For the K-12-WT, Δ*ompF,* Δ*ompC,* Δ*entB,* and Δ*fepA* strains, MICs of FDC increased between four- and eightfold, reaching respective final values varying from 0.31 to 0.5 mg/L under 100 mg/L iron concentrations and showed similar evolution (regression slopes of 0.60, 0.49, 0.51, 0.59, 0.72, respectively). The Δ*cirA* mutant showed the most marked increase in FDC MIC, rising 12-fold to reach 3 mg/L at 50 mg/L iron, with a regression slope of 0.76. The FDC MIC of the Δ*fiu* mutant increased threefold, from 0.19 to 0.5 mg/L (regression slope = 0.38). In contrast, the Δ*fecA* mutant showed a limited increase (regression slope = 0.20), with MICs remaining stable at 0.03 mg/L up to 5 mg/L and slightly increasing to 0.09 mg/L at 100 mg/L (Fig. 2A and Table S3).

For 536-PAI-deleted mutants, no differences in MIC were observed compared to 536-WT strain, trends with regression slopes ranging from 0.95 to 1.15 vs 1.07 (Fig. 2B and Table S3).

### MICs of FDC on NoBL/TEM-Ec and NDM-*Ec* clinical isolates and prevalence of iron uptake systems

Among the 89 clinical isolates, 13 NoBL/TEM-*Ec* isolates (10 with a truncating mutation in *cirA*, 1 with a truncating mutation in *fiu*, and 2 with *fiu* disrupted by an insertion sequence) and 1 NDM-*Ec* isolate (with a truncating mutation in *cirA*) had an altered CirA or Fiu receptor and were excluded from further analysis to avoid any bias related to these well-established FDC resistance determinants.

**Fig 3:**
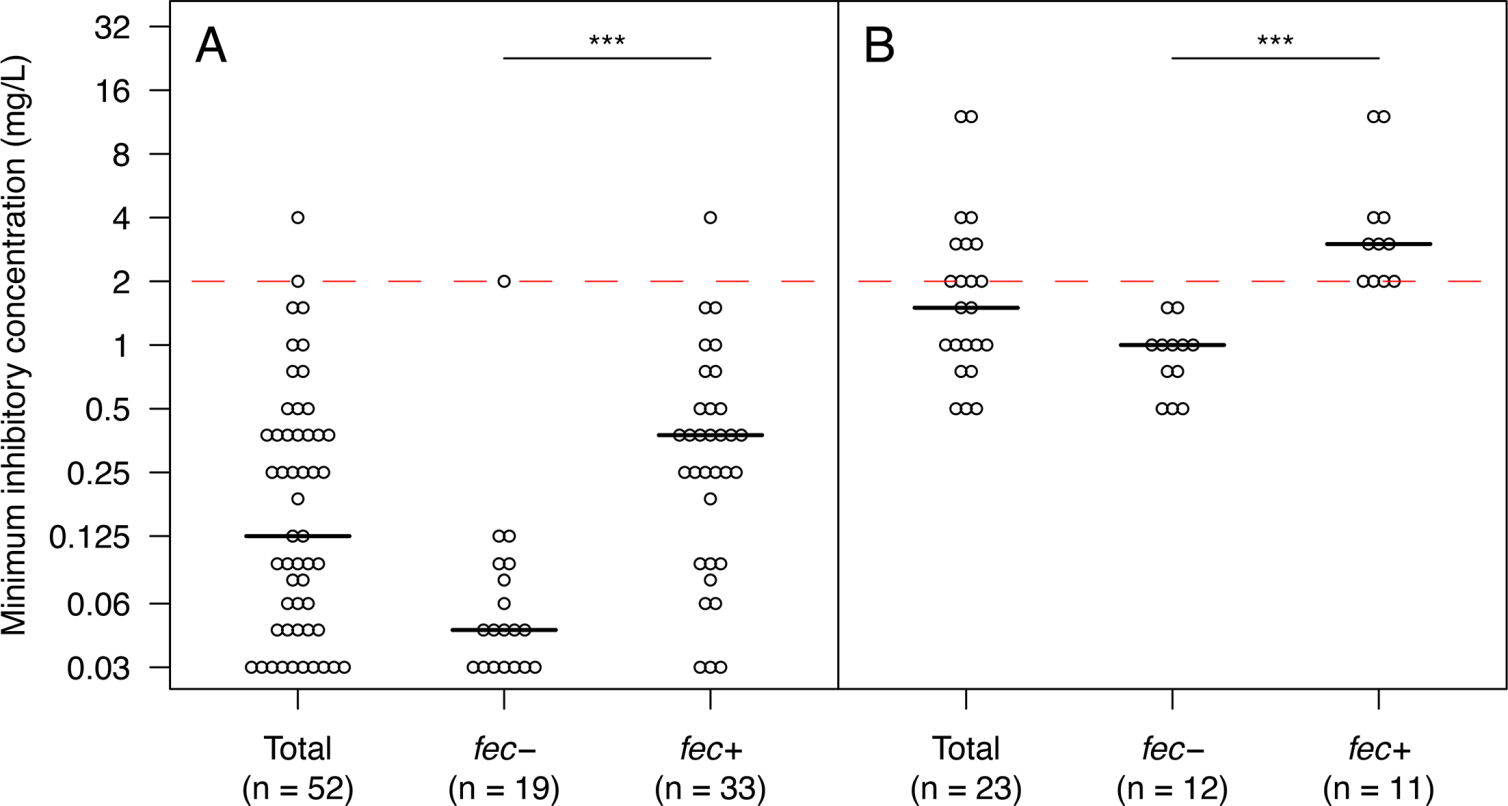
Distribution and median (horizontal line) of FDC MICs among all, *fec*-positive and *fec*-negative isolates from the two collections of *E. coli* clinical isolates: no-acquired β-lactamase or TEM-producing *E. coli* (NoBL/TEM-*Ec*) (**A**) and NDM-producing *E. coli* (NDM-*Ec*) (**B**); FDC breakpoint threshold from EUCAST (= 2 mg/L) indicated with a dotted line. *** indicates *P* < 0.001 according to a Wilcoxon test.

The FDC MICs of the 52 remaining NoBL/TEM-*Ec* isolates ranged from 0.03 to 4 mg/L with a median of 0.125 mg/L. The FDC MICs of the 23 remaining NDM-*Ec,* ranged from 0.5 to 12 mg/L with a median of 1.5 mg/L (Fig. 3). No significant difference in FDC MICs was observed based on the NDM-encoding plasmid. According to EUCAST breakpoint (2 mg/L), 98% (51/52) of the NoBL/TEM-*Ec* strains and 70% (16/23) of the NDM-*Ec* were categorized as susceptible to FDC.

The prevalence of the 13 iron uptake systems in NoBL/TEM-*Ec* and NDM-*Ec* isolates is presented in Table 1. For each group, their prevalence was compared between isolates with FDC MICs ≤ median or > median. The only iron uptake system significantly associated with higher MICs in both NoBL/TEM-*Ec* and NDM-*Ec* was the *fec* operon. Its prevalence differed from 96% (24/25) to 33% (9/27) in NoBL/TEM-*Ec* isolates (*P* < 0.001) with MICs above and below the median, respectively, and from 100% (11/11) to 0% (0/12) in NDM-*Ec* (*P* < 0.001) (Table 1). The median MIC of *fec*-positive vs *fec*-negative isolates were 0.38 and 0.05 mg/L (*P* = 0.005) for NoBL/TEM-*Ec,* respectively, and 3 and 1 mg/L for NDM-*Ec* (*P* = 0.005), respectively (Fig. 3). According to the EUCAST breakpoint, 63.6% (7/11) of *fec*-positive NDM-*Ec* were resistant to FDC, while none of the *fec*-negative were (*P* = 0.001). The aerobactin system (*iuc/iut*) was also more prevalent in NoBL/TEM-*Ec* with MIC above the median (64%, 16/25) than among those below (22%, 6/27) (*P* = 0.004). However, this association appeared to reflect co-occurrence with the *fec* operon as 95.7% (22/23) of *iuc*-positive isolates also carried the *fec* operon. Indeed, a higher MIC was consistently associated with the presence of *fec*, regardless of the presence of *iuc* (*P* < 0.05). Moreover, no significant difference in MIC distribution was observed between *fec+/iuc−* and *fec+/iuc*+ or *fec−/iuc*+ and *fec−/iuc*− isolates, in either the NoBL/TEM-*Ec* or NDM-*Ec* (Fig. S1).

**TABLE 1:** Prevalence of iron uptake systems in the two collections of *E. coli* clinical isolates (NoBL/TEM-*Ec* and NDM-*Ec*)^*a*^

	Cefiderocol MICs (mg/L)							
Genes/gene operon	NoBL/TEM-*Ec*				NDM-*Ec*			
	Total	≤0,125 mg/L *n* (%)	>0,125 mg/L *n* (%)	*P*-value	Total	≤1.5 mg/L *n* (%)	>1.5 mg/L *n* (%)	*P*-value
	*n* (%)				*n* (%)			
Strains total *n* (%)	52	27/52 (52)	25/52 (48)		23	12/23 (52)	11/23 (48)	
Heme transport systems								
*chu*	30/52 (58)	18/27 (67)	12/25 (48)	0.261	9/23 (39)	6/12 (50)	3/11 (27)	0.400
*hma*	11/52 (21)	6/27 (22)	5/25 (20)	1.000	6/23 (26)	4/12 (33)	2/11 (18)	0.640
Ferric transport systems								
TonB complex (*tonB*/*exbB*/*exbD*)	52/52 (100)	27/27 (100)	25/25 (100)	1.000	23/23 (100)	12/12 (100)	11/11 (100)	1.000
Siderophore systems								
Enterobactin (*ent*/*fep*/*fiu*/*cirA*)	52/52 (100)	27/27 (100)	25/25 (100)	1.000	23/23 (100)	12/12 (100)	11/11 (100)	1.000
Salmochelin (*iro*)	26/52 (50)	11/27 (41)	12/25 (48)	0.780	10/23 (43)	4/12 (33)	6/11 (55)	0.414
Aerobactin (*iuc*/*iutA*)	22/52 (42)	6/27 (22)	16/25 (64)	0.004	7/23 (30)	2/12 (17)	5/11 (46)	0.193
Yersiniabactin (*irp*/*ybt*/*fyuA*)	25/52 (48)	13/27 (48)	12/25 (48)	1.000	11/23 (48)	5/12 (42)	6/11 (55)	0.684
Putative siderophore system (*ire*)	7/52 (13)	4/27 (15)	3/25 (12)	1.000	2/23 (9)	0/12 (0)	2/11 (18)	0.217
Putative siderophore system (*fit*)	28/52 (54)	17/27 (63)	11/25 (44)	0.266	7/23 (30)	5/12 (42)	2/11 (18)	0.371
Iron transporters								
Citrated- base hydroxamate (*fhu*)^*b*^	52/52 (100)	27/27 (100)	25/25 (100)	1.000	23/23 (100)	12/12 (100)	11/11 (100)	1.000
Di-citrate (*fec*)	33/52 (63)	9/27 (33)	24/25 (96)	<0.001	11/23 (48)	0/12 (0)	11/11 (100)	<0.001
Ferrous transporter systems								
*efe*	51/52 (98)	27/27 (100)	24/25 (96)	1.000	23/23 (100)	12/12 (100)	11/11 (100)	1.000
*feo*	52/52 (100)	27/27 (100)	25/25 (100)	1.000	23/23 (100)	12/12 (100)	11/11 (100)	1.000
*sit*	34/52 (65)	17/27 (63)	17/25 (68)	0.776	16/23 (70)	9/12 (75)	7/11 (64)	0.667
Non-specific porins								
*ompF*	51/52 (100)	27/27 (98)	24/25 (96)	1.000	23/23 (100)	12/12 (100)	11/11 (100)	1.000
*ompC*	51/52 (100)	27/27 (98)	24/25 (96)	1.000	23/23 (100)	12/12 (100)	11/11 (100)	1.000

^*a*^ Isolates of each collection were divided into two groups according to their FDC MIC relative to the median MIC of the corresponding collection.

^*b*^ FhuA proteic sequence was screened in addition to its gene sequence as the latter is less conserved than other screened gene.

**Fig 4:**
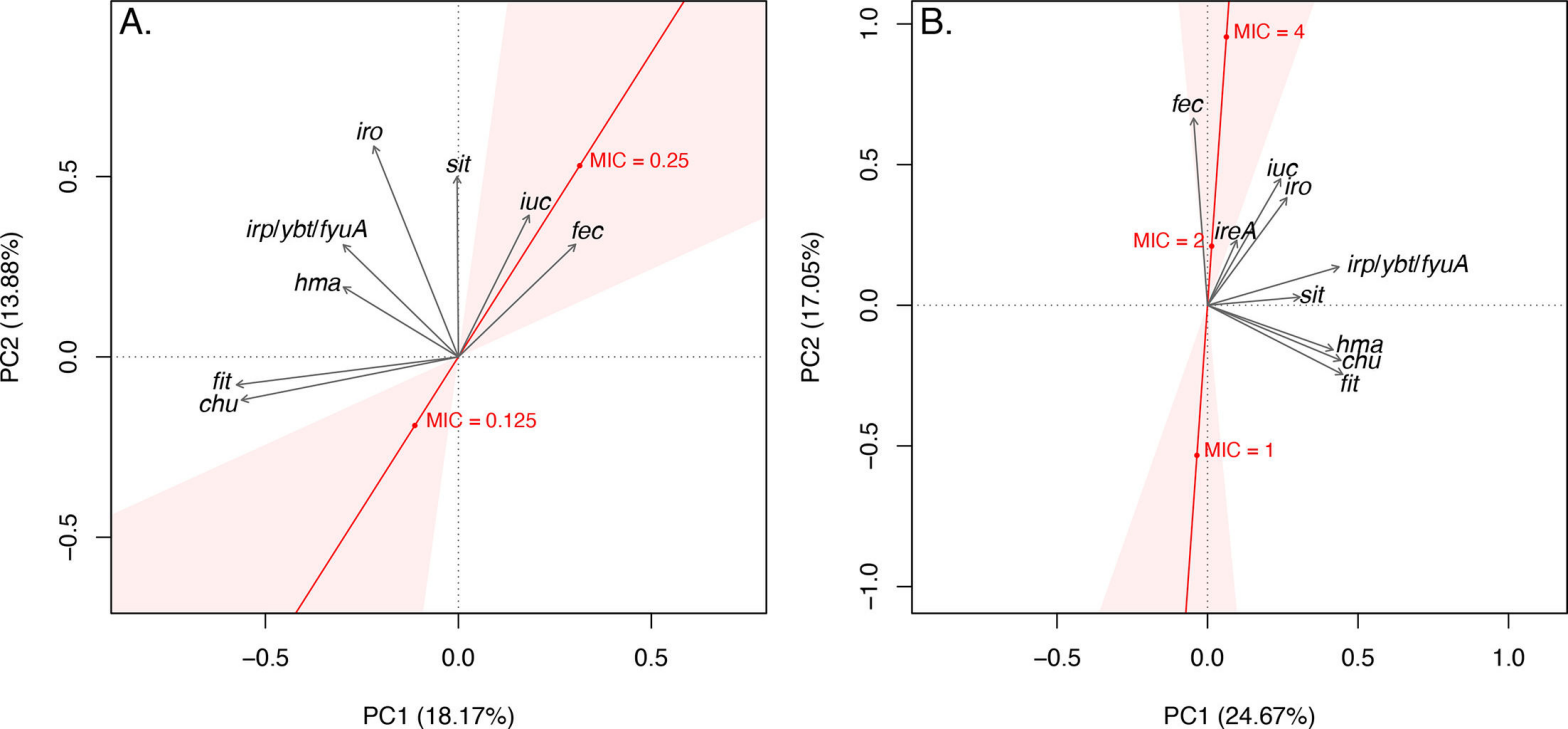
Correlation between the presence of operons and the first two axes (gray arrows) of the principal component analysis of the NoBL/TEM-*Ec* (**A**) and the NDM-*Ec* (**B**) collections, together with the predicted variation of MIC with those two axes (red line), with the 95% confidence interval of the prediction (red envelope). The MIC at the (0,0) coordinate is the average over the whole collection, and the red dots represent the position on the prediction line of measured MIC relative to this average.

A PCA was then performed to assess the relationship between operon presence and MIC of FDC. More than 90% of the variance in operon presence in the NoBL/TEM-*Ec* and NDM-*Ec* was, respectively, explained by the first nine (91.15% of variance) and eight (92.81% of variance) principal components (PC) of the PCA, which were used as factors for the MIC regressions. For the analysis of the NoBL/TEM-*Ec* collection, the significant factors were PC1 (95%IC = [0.202, 1.016], *P* < 0.004), PC2 (95%IC = [0.495, 1.559], *P* < 0.001), PC3 (95%IC = [0.229, 1.382], *P* = 0.007), PC5 (95%IC = [0.207, 1.651], *P* = 0.013), and PC6 (95%IC = [0.382, 2.132], *P* = 0.006), which were all positively correlated with the Log_2_ of MIC (Table S4). For the analysis of the NDM-*Ec* collection, the significant factors were PC2 (95%IC = [0.993, 1.685], *P* < 0.001), PC4 (95%IC = [0.071, 1.172], *P* = 0.033), PC5 (95%IC = [0.742, 1.994], *P* < 0.001), and PC7 (95%IC = [0.526, 2.382], *P* = 0.004), which were all positively correlated with the Log2 of MIC (Table S5). In both cases, the *fec* operon was the only one whose presence was positively correlated with all the significant components (Tables S6 and S7). For both collections, the changes in MIC predicted by the model are, therefore, highly correlated with the presence of the *fec* operon in the isolates (Fig. 4).

### Prevalence and genomic location of *fec* iron uptake system in *E. coli*

**Fig 5:**
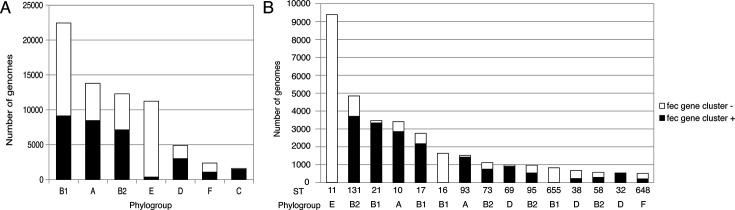
Distribution of the *fec* gene operon among *E. coli* genomes from Enterobase according to their phylogroup (**A**) and ST (**B**). Only the 15 most prevalent STs are represented in (**B**). The number of *fec* gene operon positive and negative genomes are represented by a black bar and a white bar, respectively.

By screening 70,301 *E. coli* genomes available from EnteroBase (17), 46.8% (32,905/70,301) were found to harbor the *fec* operon (*fecIRABCDE*). The prevalence of this operon varied by phylogroup, ranging from 3.2% (355/11,173) in phylogroup E to 92.5% (1,481/1,601) in phylogroup C (Fig. 5A).

Considering the 15 most prevalent sequence types (STs) in the database, the prevalence of *fec* ranged from 0.1% to 99.4%, with high prevalence observed in both virulent and epidemiologically significant clones from phylogroup B2, such as ST131 (77.0%, 3,729/4,840) and ST95 (55.6%, 545/980), as well as in commensal-associated clones such as ST10 (84.9%, 2,891/3,404) (Fig. 5B).

We then focused on a collection of complete genomes from the RefSeq database for the determination of the location of the *fec* operon. Among the 2,302 complete *E. coli* genomes studied, 48.5% (1,116/2,302) were found to harbor the *fec* operon. Most of these operons (99.1%, 1,105/1,116) were located on the chromosome and inserted within a variable genomic region. The complete operon (*fecIRABCDE*) was present in 99.1% of the chromosome-borne *fec* (1,095/1,106) and 72.7% of the plasmid-borne *fec* (8/11). The majority of incomplete operons contained only the *fecA* gene. Analysis of the eight complete plasmids did not reveal a specific incompatibility (Inc) group associated with the presence of *fec* although half of them (4/8) carried the IncFIA replicon.

Additionally, sequences generated by nanopore technology were available for 3 of the 12 *fec*-positive NDM-*Ec* isolates, enabling analysis of the genomic location of the operon. This *fec* operon was plasmid-located and complete in only a single isolate. In the remaining two strains, the *fec* operon was located on the chromosome.

Further analysis of plasmid-borne *fec* operons suggested distinct origins (Fig. S2). The *fecA* gene from our NDM-*Ec* isolate and three plasmids in *E. coli* RefSeq genomes (NZ_CP060942.1, NZ_CP021680.1, NZ_CP026937.1) were closely related to plasmid-borne *fecA* in *Klebsiella pneumoniae* (20). In contrast, *fecA* from other *E. coli* plasmids (*n* = 5) were more closely related to chromosomal *fecA* sequences of *Enterobacter hormaechei*.

## DISCUSSION

In this study, we investigated the role of various *E. coli* iron transport systems on the activity of FDC, using mutants lacking iron capture genes from well-characterized collections of *E. coli* K-12 (Keio knockout collection) and the uropathogenic *E. coli* 536 (536-PAI-deleted collection) as well as genome-sequenced clinical isolates exhibiting different β-lactam phenotypes susceptibility. The latter were either wildtype or producers of TEM penicillinase (NoBL/TEM-*Ec*) or of NDM-1 carbapenemase (NDM-*Ec*). We confirmed that the two major entry pathways for FDC are the enterobactin receptors CirA and Fiu. The presence of other siderophore such as salmochelin or yersiniabactin had no impact on FDC activity. Interestingly, the presence of the ferric citrate transporter Fec was associated with increased MICs to FDC and contributed to resistance in NDM-producing *E. coli*.

*E. coli* possesses a wide array of iron uptake systems, including heme transporters (Chu and Hma), ferrous iron transport systems (Sit, Feo, Efe) associated with non-specific porins (OmpF and OmpC), high-affinity ferric transporters (Fhu, Fec, Kfu), and siderophore systems (enterobactin, salmochelin, aerobactin, and yersiniabactin). These siderophore systems, also considered virulence factors, play a central role in iron acquisition under iron-limited conditions (21, 22). The catecholate enterobactin siderophore is ubiquitous in *E. coli* and enables Fe^3+^ uptake through its membrane receptors FepA, CirA, and Fiu. The activity of FDC is primarily linked to its catechol moiety, which mimics a siderophore and enhances its penetration into the periplasmic space of Gram-negative bacilli (23).

In our study, the FDC MIC of the Δ*cirA* and Δ*fiu* mutants, in iron-deficient media, were significantly higher than that of the K-12-WT, confirming that FDC relies on CirA and Fiu receptors to reach the periplasmic space (24, 25). These differences were even more pronounced in iron-supplemented conditions. The stronger impact of *cirA* deletion compared to *fiu* deletion is consistent with previous studies reporting that FDC has much higher affinity for CirA than for Fiu with dissociation constants (Kd) of 63 nM and 0.4 µM, respectively (24, 26). The lack of effect of *fepA* gene deletion on FDC MICs, under both iron deficient and enriched conditions, also confirmed previous findings indicating the inability of FDC to bind the FepA receptor (24). This observation aligns with known resistance mechanisms to FDC in Enterobacterales, which often involve mutations in *cirA* (8, 10, 27–29) or *fiu* (10, 27) genes, particularly truncations in *cirA* that can raise MICs higher than 32 mg/L. Mutation in the regulatory systems *baeS* and *envZ,* which control the expression of *fiu* and *cirA*, has also been associated with increased FDC MICs (29). Therefore, clinical isolates harboring truncating or disruptive mutations in *cirA* or *fiu* were excluded from all subsequent analyses.

Furthermore, although not statistically significant, we observed a slight increase in FDC MIC in the *ΔompF* mutant, whereas no change was detected in the *ΔompC* mutant. This slight increase in MIC in *ΔompF* may reflect reduced passive diffusion of FDC through outer membrane porins, as described for other β-lactams (30). The difference between the two porins likely stems from their distinct structural and physicochemical channel properties, including pore size and charge selectivity, which influence β-lactam permeation. In particular, OmpF exhibits higher permeability than OmpC for certain β-lactams such as ceftazidime (10, 28).

Notably, the deletion of *fecA*, known as a virulence factor in uropathogenic *E. coli* strains (31), led to a decrease rather than an increase in FDC MIC. This effect was more pronounced under iron-enriched conditions, where the FDC MIC of the Δ*fecA* mutant increased modestly by threefold, whereas that of the K-12 WT increased up to eightfold at the highest iron concentration. In line with these findings, the prevalence of *fec*-positive isolates was significantly higher among isolates with FDC MICs above the median than among those with MICs below the median for both NDM-*Ec* and NoBL/TEM-*Ec* collections.

These results are consistent with those of Kocer et al. who identified the *fec* operon as a contributor to FDC resistance in clinical isolates producing VIM-type carbapenemases (32). Acquisition of a *fec* operon by a VIM-producing *Citrobacter freundii* isolate resulted in at least a fourfold increase in FDC MIC, leading to phenotypic resistance. Although the presence of an MBL alone, such as VIM or NDM typically increases FDC MICs by 4 to 6 dilutions compared to wild-type strains (7, 32), MICs generally remain below the EUCAST clinical breakpoint. However, the combination of MBL production and *fec* carriage may result in a substantial increase in FDC MIC, exceeding the clinical susceptibility threshold.

**Fig 6:**
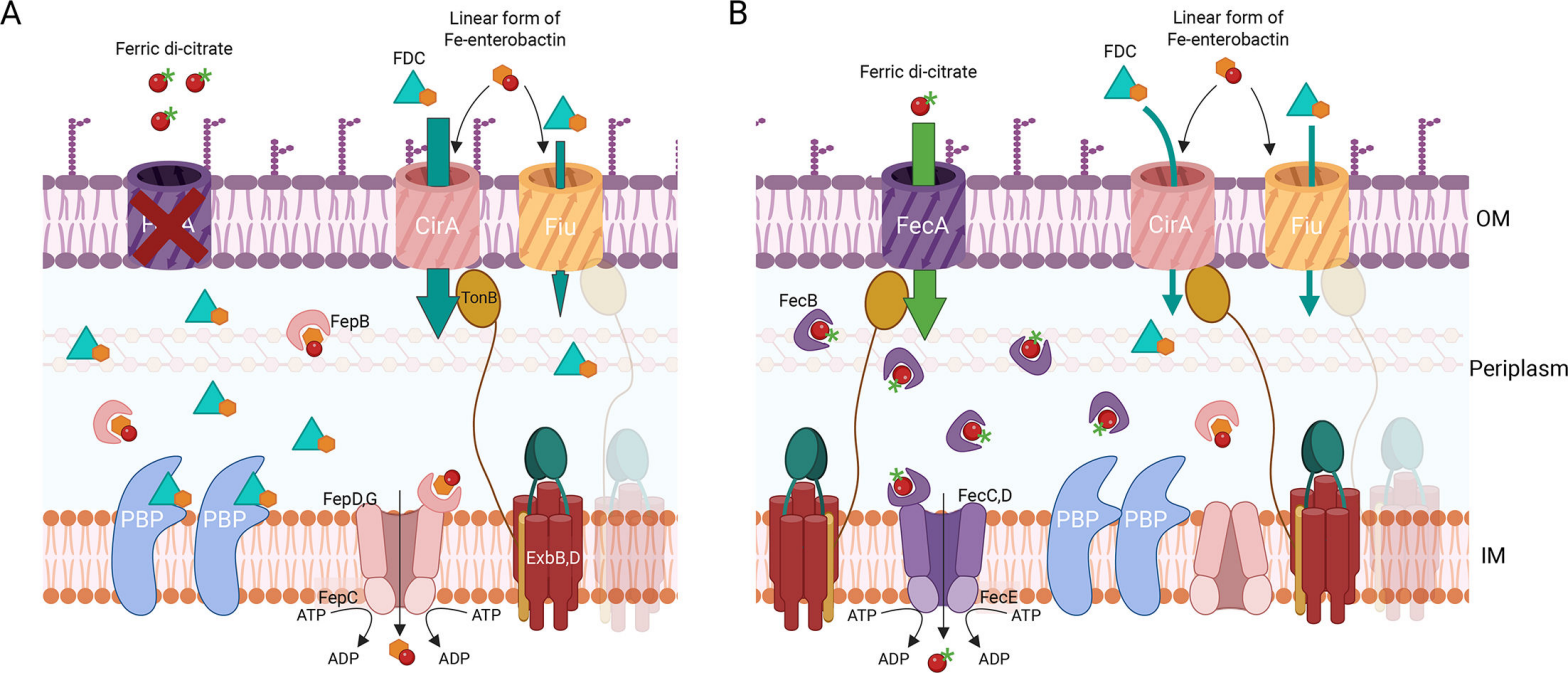
Schematic representation of the impact of the *fecIRABCDE* operon and Cefiderocol (FDC) activity. (**A**) Preferential iron uptake *via* the enterobactin (Ent) system in strains lacking the *fecIRABCDE* operon enabling efficient FDC entry (low MIC). (**B**) Ferric di-citrate uptake *via* the FecA transporter in strains carrying the *fecIRABCDE* operon, leading to increased intracellular iron concentration and downregulation of *cirA* and *fiu,* resulting in reduced use of the enterobactin uptake system and decreased FDC entry (higher MIC). Abbreviations: Outer membrane (OM), Inner membrane (IN), Penicillin binding protein (PBP). Created in BioRender. Armand, L. (2026) https://BioRender.com/jaj9e83 (License XT29NR0HD6).

To explain these observations, Polani et al. assessed the expression of siderophore receptor genes in the presence and absence of a plasmid-borne *fec* operon and ferric citrate in an *E. coli* K-12 model. They reported a significant decrease in the expression of *fiu*, *cirA*, *fepA*, and *fhuA* in *fec*-positive strains grown in the presence of 0.5 μM and 5.0 μM ferric citrate. They further speculated that iron imported via the plasmid-encoded Fec system was sufficient to downregulate the expression of these TonB-dependent receptors, which are also involved in FDC uptake (20) (Fig. 6). In contrast, Kocer et al. did not observe reduced expression of enterobactin receptors; however, their experiments were not conducted under ferric citrate-supplemented conditions.

Overall, the *fec* operon is widespread in *E. coli,* present in nearly half (46.5%) of the 70,301 strains from Enterobase screened in this study. It is found in both epidemiologically significant virulent clones, such as ST131 and ST95, and commensal-associated clones, such as ST10. While commonly located on the chromosome, it can also be found on mobile genetic elements such as plasmids. Plasmid-borne *fec* operons identified in *E. coli* genomes from the RefSeq database were closely related either to chromosomal operon of *Enterobacter hormaechei* (*n* = 5) and *Klebsiella pneumoniae* (*n* = 3) or, as observed in one of our NDM-producing isolates, to the recently described plasmid-borne operon of *K. pneumoniae* (20). These findings suggest mobilization of chromosomal *fec* genes and transfer onto plasmids although an initial plasmid origin followed by chromosomal integration cannot be formally excluded. They also highlight the subsequent dissemination of such plasmids across different bacterial species. Consistent with this, in the study by Kocer et al. (32), *fec* was carried by a broad host-spectrum plasmid, transferable by conjugation to other Enterobacterales but also to *Pseudomonas aeruginosa*. The presence of the *fec* operon on transferable genetic elements poses an additional threat to FDC efficacy on carbapenemase producing strains as FDC remains a last-line therapeutic option.

The deletion of the other siderophore systems, salmochelin and yersiniabactin, investigated using 536-PAI-deleted collection, had no impact on FDC MICs in either iron-depleted or iron-supplemented conditions suggesting that there is no competition between those siderophore systems and the enterobactin system. The higher prevalence of the aerobactin system (*iuc*) among NoBL/TEM-*Ec* isolates with FDC MICs above the median MIC appears to result from a frequent co-occurrence with the *fec* operon. This association was not observed in the NDM-*Ec* collection, and no difference in MIC was found between *fec*-positive/*iuc*-negative and *fec*-positive/*iuc*-positive isolates (Fig. S1). Finally, no additional associations between FDC susceptibility and the prevalence of iron uptake genes were observed among the collection of clinical isolates.

From an evolutionary perspective, our data provide further evidence to support the molecular mechanism involving iron that breaks the classical trade-off between virulence and antibiotic resistance (33) as the strains acquiring *fec* are both more virulent and more resistant. These data are an additional illustration of the many situations in which iron and antibiotics have combinatorial effects when used together, sometimes referred to “liaisons dangereuses” (34).

Our study has several limitations. First, all mutants used (except ΔPAI-III and PAI-dTTP) were single-gene deleted for iron uptake genes, which prevents the evaluation of potential additive or synergistic effects between iron uptake systems, particularly in combination such as *cirA*/*fiu*, *fec*/*cirA,* or *fec*/*fiu* double mutants. Second, we determined only FDC MIC and did not perform time-killing assays, as conducted in other studies (35). Finally, we did not directly assess gene expression or protein levels of iron transport systems. However, the regulatory architecture governing iron homeostasis is conserved between *K. pneumoniae* and *E. coli*, including the Fur regulator, the catecholate receptors (*cirA*, *fiu*), and the *fec* operon. This supports that the mechanism described by Polani et al. (20) in *K. pneumoniae* should be the same for *E. coli*.

### Conclusion

In addition to confirming the role of the CirA and Fiu receptors, our study highlights the ferric citrate transporter Fec as a novel resistance determinant affecting FDC activity in *E. coli*. This is particularly concerning given its prevalence in nearly 50% of *E. coli strains*, its potential for plasmid-mediated transfer, and its ability to confer FDC resistance when combined with other mechanisms, such as MBL production. By linking increased virulence and resistance, the *fec* operon exemplifies how iron acquisition can override the classical trade-off between pathogenicity and antibiotic susceptibility.

## SUPPLEMENTAL MATERIAL

Supplemental figures aac.01679-25-s0001.docx Fig. S1 and S2.

Supplemental methods aac.01679-25-s0002.docx Description of the sequencing of the NDM-Ec isolates and analysis of the relevant virulence genes.

Supplemental tables aac.01679-25-s0003.xlsx Tables S1 to S7.

ASM does not own the copyrights to Supplemental Material that may be linked to, or accessed through, an article. The authors have granted ASM a non-exclusive, world-wide license to publish the Supplemental Material files. Please contact the corresponding author directly for reuse.
