# Supplementary material for: Influence of iron uptake systems on cefiderocol activity in Escherichia coli: At the crossroads of antibiotic resistance and virulence
Source: Antimicrob Agents Chemother. 2026 Jul 10;70(8):e01679-25. doi: 10.1128/aac.01679-25 (PMC13436293; doi:10.1128/aac.01679-25)
Supplement: Supplementary file 1 [file aac.01679-25-s0001.docx]

**Figure S1:** Distribution and median (horizontal line) of FDC MICs among, *fec*-negative/*iuc*-negative (*cf.* Figure 3), *fec*-positive/*iuc*-negative, *fec*-negative/*iuc*-positive, and *fec*-positive/*iuc*-positive isolates from the two collections of *E. coli* clinical isolates: no acquired β-lactamase or TEM-producing *E. coli* (NoBL/TEM-*Ec*) (A) and NDM-producing *E. coli* (NDM-*Ec*) (B). * and *** indicate p < 0.05 and p < 0.001 respectively according to a Wilcoxon test (P values corrected by Bonferroni-Holm method).

**Figure S2:** Physical maps of *fec*-carrying plasmids from our *E. coli* NDM-producing isolate, *E. coli* RefSeq genomes, and *Klebsiella pneumoniae*. The *fec* operon is highlighted in red, and surrounding insertion sequences in pink. Other coding sequences homologous between maps are represented by colored arrows. The grayscale of the links between maps indicates the level of protein identity (minimum identity: 60%). Plasmid names, accession numbers, and bacterial species from which the plasmid was isolated are indicated on the left side of the maps.
