# Supplementary material for: Influence of iron uptake systems on cefiderocol activity in Escherichia coli: At the crossroads of antibiotic resistance and virulence
Source: Antimicrob Agents Chemother. 2026 Jul 10;70(8):e01679-25. doi: 10.1128/aac.01679-25 (PMC13436293; doi:10.1128/aac.01679-25)
Supplement: Supplementary file 2 [file aac.01679-25-s0002.docx]

**Supplementary methods**

As part of the BCB bacteriology laboratory's activity, clinical strains were sequenced and analyzed as described previously (14). Briefly, bacterial DNA was extracted using the EZ1 DNA tissue kit (Qiagen, Hilden, Germany) on the EZ1 Advanced XL device (Qiagen). After the determination of DNA concentrations with with Invitrogen™ Qubit™ 4 Fluorometer with NanoDrop 2000c (Thermo Fisher Scientific, Waltham, MA, USA), DNA extracts were traited with the Illumina DNA Prep kit Illumina^®^ (San Diego, CA, USA, then sent to LGC Group (LGC Genomics, Berlin, Germany), for library preparation and sequencing. Then, WGS was achieved on a NextSeq Miniseq platform, Illumina^®^ (San Diego, CA, USA).

Analysis of raw Illumina reads were executed after used of Trimmomatic v0.39 and Trim Galore v0.6.7. Raw and trimmed reads quality were checked with FastQC v0.11.9. Metaphlan v4.0 was used to verify identifications of our strains and to look for putative cross-contaminations. Reads were assembled by using SPAdes v 3.15.4 and the quality of assemblies was examined by using QUAST v5.0.2. Resistance and virulence genes were searched by using Abricate v1.0.0 using ResFinder4 (coverage and identification cut-off fixed at 90% and 70% respectively) and custom database respectively. This database included gene related to iron uptake system: *chuA*, *chuS*, *chuT*, *chuV*, *chuW*, *chuX*, *chuY*, *hmA*, *entA*, *entB*, *entC*, *entD*, *entE*, *entF*, *entH*, *entS*, *cirA*, *fiu*, *fepA*, *fepB*, *fepC*, *fepD*, *fepE*, *fepG*, *fes*, *ireA*, *iroB*, *iroC*, *iroD*, *iroE*, *iroN*, *irp1*,*irp2*, *ybtA*, *ybtD*, *ybtE*, *ybtP*, *ybtQ*, *ybtS*, *ybtT*, *ybtU*, *ybtX*, *fyuA*, *iucA*, *iucB*, *iucC*, *iucD*, *iutA*, *fhuA*, *fhuB*, *fhuC*, *fhuD*, *fhuE*, *fhuF*, *fecA*, *fecB*, *fecC*, *fecD*, *fecE*, *fecI*, *fecR*, *ompC*, *ompF*, *efeB*, *efeO*, *efeU*, *feoA*, *feoB*, *feoC*, *sitA*, *sitB*, *sitC*, *sitD*, *tonB*, *exbB*, *exbD*, *baeS*, *envZ*, *fetA*, *fetB*, *fitA*, *fitB*, *fitC*, *fitD*, *fitE* and *fitR*. Most of the sequence were found in NCBI database (https://www.ncbi.nlm.nih.gov) from *Escherichia coli* K-12 (MG1655) or from five other *E. coli* strains: *hma* (or C2482(33,34)) from *E. coli* O6:H1 (strain CFT073 / ATCC 700928/ UPEC), *sitD* from *E. coli* plasmid pAPEC-O2-ColV, *ireA* from *E. coli* ED1a, *fitA* and *efeU* from *E. coli* O157:H7 (Sakai). Others genes from the *fit* operon are from Ouyang *et al.* previous study (Infection and Immunity, 2006), indexed in NCBI. Some sequences came from other Enterobacterales: *irp1*, *irp2*, *ybtA*, *ybtE*, *ybtP*, *ybtQ*, *ybtS*, *ybtT*, *ybtU*, *ybtX* and *fyuA* from Yersinia pseudotuberculosis (IP 32953), *ybtD* from Yersinia pestis A1122, *iroB*, *iroC*, *iroD*, *iroE*, *iroN*, *sitA*, *sitB* and *sitC* from *Salmonella enterica subsp. enterica serovar Typhimurium* (str. LT2). The sequences of *sitABC* and *hma* were found in the UniProt database (https://www.uniprot.org). Please refer to their accession numbers provided in Table S1. PlasmidFinder v2.0.1 was used to detect plasmid replicons
